# Comparison of Current Surgical and Non-Surgical Treatment Strategies for Early and Locally Advanced Stage Glottic Laryngeal Cancer and Their Outcome

**DOI:** 10.3390/cancers12030732

**Published:** 2020-03-20

**Authors:** Olgun Elicin, Roland Giger

**Affiliations:** 1 Department of Radiation Oncology, Inselspital, Bern University Hospital, University of Bern, CH-3010 Bern, Switzerland; olgun.elicin@insel.ch; 2 Department of Otorhinolaryngology, Head and Neck Surgery, Inselspital, Bern University Hospital, University of Bern, CH-3010 Bern, Switzerland

**Keywords:** laryngeal cancer, head and neck cancer, squamous cell carcinoma, surgery, radiotherapy, organ-preservation, chemotherapy

## Abstract

For the treatment of early and locally advanced glottic laryngeal cancer, multiple strategies are available. These are pursued and supported by different levels of evidence, but also by national and institutional traditions. The purpose of this review article is to compare and discuss the current evidence supporting different loco-regional treatment approaches in early and locally advanced glottic laryngeal cancer. The focus is kept on randomized controlled trials, meta-analyses, and comparative retrospective studies including the treatment period within the last twenty years (≥ 1999) with at least one reported five-year oncologic and/or functional outcome measure. Based on the equipoise in oncologic and functional outcome after transoral laser surgery and radiotherapy, informed and shared decision-making with and not just about the patient poses a paramount importance for T1-2N0M0 glottic laryngeal cancer. For T3-4aN0-3M0 glottic laryngeal cancer, there is an equipoise regarding the partial/total laryngectomy and non-surgical modalities for T3 glottic laryngeal cancer. Patients with extensive and/or poorly functioning T4a laryngeal cancer should not be offered organ-preserving chemoradiotherapy with salvage surgery as a back-up plan, but total laryngectomy and adjuvant (chemo) radiation. The lack of high-level evidence comparing contemporary open or transoral robotic organ-preserving surgical and non-surgical modalities does not allow any concrete conclusions in terms of oncological and functional outcome. Unnecessary tri-modality treatments should be avoided. Instead of offering one-size-fits-all approaches and over-standardized rigid institutional strategies, patient-centered informed and shared decision-making should be favored.

## 1. Introduction

Laryngeal squamous cell carcinomas comprise around 25% of all head and neck cancers [1]. The major predisposing factor is tobacco and alcohol abuse, which is declining over the past years [2] thanks to public awareness.

For the treatment of early and advanced glottic laryngeal cancer, multiple strategies are available. These are pursued and supported by different levels of evidence, but also by national and institutional traditions. Patients’ individual preferences, social, cultural and economic backgrounds play a major role in the chosen treatment approach as well. The main debate regarding the treatment of UICC (Union for International Cancer Control) stage I-II glottic laryngeal cancer (T1-2N0) is about the optimal selection of a unimodal treatment (i.e., surgery or radiotherapy). On the other hand, the discussion of advanced glottic laryngeal cancer (T3-4aNanyM0) is mostly about the definition of ideal algorithms concerning the sequence and combination of multi-modal strategies. The delicate tradeoff between oncologic outcome and quality of life requires a more careful and individualized decision-making process in patients with advanced tumors.

The purpose of this review article is to compare and discuss current evidence supporting surgical and non-surgical loco-regional treatment approaches in early and advanced glottic laryngeal cancer, except for the cT4b primaries and recurrent or distant metastatic disease. The debate about the ideal treatment modality of laryngeal cancer is quite old [3,4]. Therefore, focus is kept on randomized controlled trials, meta-analyses and comparative retrospective studies in the treatment period of the last twenty years (≥ 1999) with at least one reported five-year oncologic and/or functional outcome measure comparing surgical and non-surgical treatment strategies. Due to the lack of robust data, cost-effectiveness is not addressed in this review.

## 2. Early Stage Glottic Laryngeal Cancer

### 2.1. T1N0

The main discussion of early stage glottic laryngeal cancer focuses on the optimal treatment modality to achieve best local control and functional outcome, the latter being restricted with voice quality. Other functional consequences, such as impaired swallowing and risk of regional or distant metastases, are quite rare.

#### 2.1.1. Oncologic Outcome

Studies comparing transoral laser surgery and radiotherapy in the treatment of T1N0 glottic laryngeal cancer within the last 20 years report comparable oncologic outcomes; namely local control, disease-specific, disease-free and overall survival (except for one study demonstrating superior local control and disease-free survival after radiotherapy [5] in a mixed cohort of T1a and T1bN0 tumors), despite institutional and personal selection biases (Table 1). Previously published meta-analyses [6,7,8,9,10,11,12] were not restricted to the treatment period defined in this article (≥ 1999). Nevertheless, most of their findings corroborate the results demonstrated here. Two recent meta-analyses [10,11] report better overall survival after transoral laser surgery than radiotherapy, despite being able to show any difference in local control. Considering the patterns of recurrence and high success rates of salvage treatments [13] in the early stage glottic laryngeal cancer, these findings support the presence of selection bias due to terms of competing risks for death in the retrospectively collected data.

Although partial open laryngectomy may be indicated in T1 glottic laryngeal cancer, this technique has become less popular in the current surgical treatment strategies since the introduction of transoral laser surgery. We are only aware of one old, randomized trial, that compared open surgery and radiotherapy in patients recruited between 1979 and 1987 [22]. Five-year, disease-free and overall survival were 71.1% vs. 100% and 91.7% vs. 100% following radiotherapy and surgery, respectively. There were no significant differences in the oncologic outcome between the two groups. These results are comparable with the outcome of the studies showed in Table 1. No other comparison studies were found in the current literature.

#### 2.1.2. Voice Quality and Laryngeal Preservation

The measures used to assess and compare voice quality after transoral laser surgery and radiotherapy are quite heterogeneous. Most reports indicate better voice quality in various domains after radiotherapy compared to transoral laser surgery [14,16,19,20] (Table 1). This effect correlates with the extent of cordectomy [20]. Despite having included a treatment period starting before 1999 (1998–2008) and being closed due to slow accrual (*n* = 60), it is worth mentioning the only randomized controlled trial comparing transoral laser surgery and radiotherapy for T1aN0 glottic laryngeal cancer, with the primary endpoint being expert-rated voice quality [23]. Overall voice quality was comparable between the two arms, but the glottal gap was wider and the voice was breathier in the surgical arm. Patients undergoing surgery reported more hoarseness-related inconvenience two years after treatment. Previously published meta-analyses were not restricted with the treatment period defined in this article (≥ 1999). However, they could not show a clinical difference comparing voice quality between transoral laser surgery and radiotherapy [7,24,25,26].

Some older retrospective studies and meta-analyses demonstrate superior laryngeal preservation after primary surgery compared to radiotherapy [7,9,10,11]. However, this finding is not seen in any modern series (Table 1). In this regard, the following aspects must be taken into consideration: (1) Due to a high selection bias in terms of co-morbidity, age, smoking and tumor extent in the retrospectively collected data, it is impossible to come to a convincing conclusion concerning larynx preservation by comparing two treatment modalities; (2) The failure to reproduce the results of older series in the more recent analyses may be associated to the technical advances in surgery and radiotherapy [27,28], utilization of superior dose-fractionation regimens [29,30,31,32,33] and smaller radiation volumes [28,34]; and (3) The conventional reasoning in favor of primary surgery is the possibility of successful salvage radiotherapy in case of treatment failure, whereas the salvage surgery after radiotherapy failure often involves a total laryngectomy. Theoretically, this may be a valid argument. However, even if that still holds true in the modern era, approximately 20 patients have to be treated with surgery instead of modern radiotherapy in order to ultimately preserve the larynx of one patient. Finally, yet importantly, surgical strategy may lead to more re-resections in order to achieve clear margins and/or for recurrences and second primaries [15]. For some patients this may pose a significant psychological stress despite an excellent ultimate local control with surgery. On the contrary, other patients undergo psychologic burden due to “radiophobia” concerning secondary malignancies, although not backed by evidence [35,36]. Properly designed studies comparing modern surgical and radiation techniques are necessary to define optimal strategies for patients with individual characteristics and expectations.

The only randomized trial, which compared open surgery and radiotherapy in patients recruited between 1979 and 1987, did not report about voice quality and laryngeal preservation [22]. We are not aware of any other prospective or retrospective comparative studies in the literature.

### 2.2. T2N0

#### 2.2.1. Oncologic Outcome

Articles reporting on oncologic outcome of patients with T2N0 glottic laryngeal cancer treated in the last two decades are very limited (Table 1). Cömert et al. [21] published a retrospective series with 3-year outcome, which yielded comparable local control, disease-free survival and larynx preservation despite a selection bias due to different timeframes in which transoral laser surgery and radiotherapy were introduced as available techniques in the same institution. Remmelts et al. [14], reporting on mixed cohorts of T1 and T2N0 tumors, were not able to show any statistically significant difference in various oncologic endpoints. These findings are in line with non-comparative reports on T2 glottic laryngeal cancer treated with transoral laser surgery [37,38,39,40,41,42] or radiotherapy [29,31,32,43,44,45,46,47,48] only. The systematic review by Warner et al. [49] comparing transoral laser surgery and radiotherapy showed equipoise in oncologic outcome and worse outcome of tumors causing impaired mobility of vocal folds (T2b), regardless of chosen treatment modality. However, this review has a high risk of selection bias and low quality of data in terms of being able to make a robust comparison and the majority of the analyzed studies included patients treated before 1999. In addition, it is not clear why the authors decided to exclude series including surgically treated cases requiring adjuvant radiotherapy from their analysis.

Although partial open laryngectomy may be indicated in T2 glottic laryngeal cancer since the introduction of transoral laser surgery, like in T1 glottic cancers, this technique has become less popular among contemporary head and neck surgeons. We could find only one randomized trial that compared open surgery and radiotherapy in patients recruited between 1979 and 1987 [22]. Five-year disease-free and overall survival were 60.1% vs. 78.7% and 88.8% vs. 97.4% following radiotherapy and surgery, respectively. Only the five-year disease-free survival showed statistically significant difference in favor of surgery. No other comparative study was found in the current literature. Retrospective non-comparative studies about cT2 laryngeal cancer treated with open partial horizontal laryngectomy showed a five-year loco-regional control, disease-specific survival and overall survival rate of 85.7–97.5%, 85.7–98% and 75–93.1%, respectively [50,51]. These results are comparable, and even better, than the outcome of the studies comparing surgical and non-surgical treatment strategies showed in Table 1, which may be caused by a selection bias (patients in a better health and performance status, favorable tumor localisation, etc.), making any conclusions difficult.

#### 2.2.2. Voice Quality and Laryngeal Preservation

To best of our knowledge, there is no study on voice quality or larynx preservation comparing surgery and radiotherapy for purely T2 primaries performed within the last 20 years (Table 1). Two retrospective analyses which report on combined cohorts of T1 and T2N0 demonstrated lower (superiority) voice handicap index scores after transoral laser surgery compared to radiotherapy [14,19]. One of these two analyses also yielded lower (inferior) understandability score and higher likelihood of hoarseness after surgery [19]. In both studies, larynx preservation was not statistically significant different between transoral laser surgery and radiotherapy, although there was a trend for higher rate of preservation after surgery [14,19]. However, this trend may be explained by the selection bias between two treatment modalities, at least regarding T stage in the study by Remmelts et al. [14]: Tis/T1a/T1b/T2 distribution was 23/49/15/2 in the surgical (*n* = 89) group and 3/54/27/75 in the radiotherapy (*n* = 159) group (*p* < 0.001). Another retrospective study including a cohort of T1 and T2N0 glottic laryngeal cancer describes the relationship of voice quality between each type of transoral laser surgery cordectomy and showed equivalent results between type I and II cordectomy and T1N0 radiotherapy. Type III cordectomy resulted in worse voice quality compared with T1N0 irradiation. Voice quality after type IV cordectomy was similar to radiotherapy to T2 tumors [20].

The only randomized trial that compares open surgery and radiotherapy in patients recruited between 1979 and 1987 does not report about voice quality and laryngeal preservation [22]. No other comparative study was found in the literature. Retrospective non-comparative studies of cT2 laryngeal cancer treated by open partial horizontal laryngectomy show a laryngeal preservation rate of 91.2–98.5% [50,51]. These results are comparable with the outcome of the studies comparing surgical and non-surgical treatment strategies showed in Table 1.

### 2.3. Anterior Commissure Involvement

Both in T1 and T2 early stage glottic cancers, the impact of the involvement of the anterior commissure on oncological outcome after different treatment modalities is a subject of debate due to partially contradicting reports [52]. Regarding surgery, anterior commissure is reported to reduce the local control after transoral laser surgery and partial laryngectomy and disease-free survival after transoral laser surgery [53,54,55,56]. On the other hand, other retrospective studies on cricohyoidoepiglottopexy show no impairment of outcome in the presence of anterior commissure involvement [57,58]. The most recent study by Allegra et al. [59] demonstrates no statistically significant differences in local recurrence-free and disease-specific survival after cricohyoidoepiglottopexy in regard to anterior commissure involvement. Regarding radiotherapy, anterior commissure involvement is reported to reduce the local control [60,61,62,63] and disease-specific survival [62]. However, reports showing no difference also exist [13,64]. In the light of low-level evidence concerning the impact of anterior commissure, no clear recommendation can be made regarding the “treatment of choice”. By mostly reviewing the previously published literature on the patients treated before 1999, Hartl et al. [52] were also not able to suggest any ideal treatment modality in the presence or absence of anterior commissure.

## 3. Locally Advanced Stage Glottic Laryngeal Cancer

### 3.1. Overall UICC Stage III–IV

Advanced stage glottic laryngeal cancer often requires a multimodal treatment concept, which usually consists of surgery and/or radiotherapy with or without chemotherapy. Main influencing factors for treatment decision are related to the primary tumor, its infiltration patterns and impact on phonation, breathing and swallowing function; as well as the patients’ preferences and expected prognosis after the chosen treatment. Although still important for the prognosis and decision on treatment intensity, the nodal stage is less important than T stage for the decision on the primary treatment modality. It is difficult to convey a high-level evidence-based discussion about the management of glottic laryngeal cancer separately on T3 and T4 primaries, because major clinical trials questioning the optimal treatment strategy did not include a homogenous population of patients. Some allowed the inclusion of advanced cases due to nodal positivity despite of T1–2 tumors, whereas others excluded “large” T4 primaries [65,66,67]. Per definition, T4b glottic laryngeal cancer implies to an inoperable tumor, and is therefore not a subject of debate in terms of optimal treatment strategies. Patients diagnosed with such extensive tumors are treated with either a combined chemoradiotherapy or a palliative approach.

As an alternative to total laryngectomy, the concept of larynx-preservation by the combination of radiation and chemotherapy emerged in the last three decades, which was strongly supported by the Veterans Affairs Laryngeal Cancer Study [67,68]. This ignited a trend to offer larynx-preserving modalities to the majority of the patients with loco-regionally advanced laryngeal cancer. However, later published data demonstrated decreased survival rates, presumably because of offering the non-surgical approach to patients, who were not carefully selected [69,70]. The majority of the head and neck oncologists later realized that the preservation of the larynx and hypopharynx as an anatomical entity does not always equate to the preservation of the organ-function and does not ensure to maintain the same quality of life in the long term. This paved the way to the definition of the composite endpoint “laryngo-esophageal dysfunction-free survival” in the consensus panel recommendations published by Lefebvre and Ang [71] 10 years ago.

Two major trials, namely the Veterans Affairs Laryngeal Cancer Study [67,68] and the Radiation Therapy Oncology Group 91-11 [66,72] defined the outcome laryngeal preservation, indeed based on whether the larynx remained in place or not. Many patients with T4 primaries were excluded from both trials. The Veterans Affairs Laryngeal Cancer Study resulted in 56% patients with T4 primaries (vs. 29% < T4, *p* = 0.001) eventually undergoing a salvage laryngectomy [67]. The two-year overall survival was not different (68% in both arms, *p* = 0.98) after primary surgery followed by adjuvant radiotherapy or induction chemotherapy followed by primary radiotherapy. Local recurrences (12% vs. 2%, *p* = 0.001) were more frequent in the primary radiotherapy arm, whereas distant metastases (17% vs. 11%, *p* = 0.001) and second primary cancers (6% vs. 2%, *p* = 0.048) were higher after the primary surgery. A later analysis on the 46 out of 65 long-term survivors of the Veterans Affairs Laryngeal Cancer Study reported better mental health, less depression and less pain among patients in the non-surgical arm [68].

Based on the findings of the Veterans Affairs Laryngeal Cancer Study, the Radiation Therapy Oncology Group 91-11 trial was designed not to include “high-volume” T4 primaries (i.e., tumor penetrating through the cartilage or extending more than 1 cm into the base of the tongue [72]), which resulted in only 10% of the accrued patients having T4 primaries [66]. In this trial, patients were randomized into three arms consisting of either radiotherapy alone, radiotherapy and concomitant cisplatin every three weeks, or two to three cycles (based on clinical response) of induction chemotherapy with cisplatin and 5-fluoruracil, followed by radiotherapy alone. The initial report of the Radiation Therapy Oncology Group 91-11 [66] showed a higher rate of intact larynx after concomitant chemoradiotherapy (88%), compared to induction chemotherapy followed by radiotherapy (75%, *p* = 0.05) and radiotherapy alone (70%, *p* < 0.001) after two years. Locoregional control was also significantly superior with concomitant chemoradiotherapy. Without any chemotherapy (radiation alone), the rate of distant metastases and disease-free survival was worse than in the other two arms with chemotherapy. However, the overall survival was comparable among all three arms. The rates of toxicities after combined regimens were around 20% higher than after radiation alone. The subsequent long-term analysis after a median follow-up of 10.8 years [72] concluded, that (1) the two combined regimens yielded similar results in laryngectomy-free survival; (2) concomitant chemoradiotherapy yielded superior locoregional control and ultimate larynx preservation; and (3) overall survival and late toxicity was comparable in all three arms. Interestingly, more deaths unrelated to laryngeal cancer occurred after concomitant chemoradiotherapy than after the induction chemotherapy and radiotherapy (70% vs. 53% within all deaths, *p* = 0.03). A recent analysis and modified representation of the Radiation Therapy Oncology Group 91-11 trial data by Licitra et al. [73] showed, how different the same data can be interpreted concerning larynx preservation. In the original analysis, deaths after total laryngectomy were ignored and higher mortality “squeezed” the cumulative incidence of total laryngectomy in the concomitant chemoradiotherapy arm (mainly referring to the Figure 2A in the report on long-term results [72]). Nevertheless, due to the lack of other statistical parameters such as confidence intervals, this critique does not prove that the concomitant approach is definitely worse than induction approach, but still suggests that it may not necessarily be superior as postulated by the study group [72]. The counterargument to the interpretation by Licitra et al. can be found in the correspondence by Forastiere et al. [74]. Finally, more importantly, it must be remembered, that the Radiation Therapy Oncology Group 91-11 trial [66] used an outdated induction chemotherapy regimen with cisplatin and 5-fluoruracil which was shown to result in inferior survival compared to docetaxel added to this doublet [75,76,77].

Regardless of the T stage, the pretreatment organ function is a significant indicator to predict the functional consequence for the patient. For patients without destruction of laryngeal cartilage scaffold, significant aspiration or problems with phonation, using the tumor response to a short course of induction chemotherapy seems to be a promising strategy to select patients, who would benefit from a non-surgical, organ preservation approach [78,79,80]. On top of an optimal strategy to select the ideal patients for organ and function preservation, establishing meticulous follow-up schedules poses paramount importance to ensure timely diagnosis of recurrences, which may be eligible for salvage surgery [81]. With passing time, salvage poses higher risks for complications such as wound dehiscence and fistula formation, need for extensive reconstruction, and prolonged hospitalization; reducing the quality of life and increasing mortality [82,83,84].

It is important to emphasize that so far, no larynx-preservation strategy results in superior survival when compared with primary surgery followed with adjuvant treatment. Nevertheless, the search to identify predictive markers to accurately define the patients who would benefit from larynx preservation with a comparable oncologic and better functional outcome continues. In addition to the above-mentioned pre-treatment anatomical criteria and tumor shrinkage after induction chemotherapy (arbitrary cut-offs usually ranging from 30–50%), there were recent efforts to develop quantifiable scoring systems predicting larynx-preservation outcome. The TALK model developed by Sherman et al. [85] uses T stage (T4), albumin (< 4 g/dL), maximum alcohol intake (i.e., Liquor) (≥ 6 cans of beer or equivalent alcohol per day or major alcohol use), and Karnofsky Performance Status (< 80%) as parameters to assign one point for each, which resulted in a total score. They used the prospectively collected data from Memorial Sloan-Kettering Cancer Center to build the model, and the Veterans Affairs Laryngeal Cancer Study data [67,68] to validate it. The authors categorized the TALK scores as good (0), intermediate (1–2) and poor risk (3–4). The three-year larynx-preservation rates (i.e., local control without any surgery to the primary site and without a permanent tracheostomy or gastrostomy) by increasing scores were 65% (TALK score = 0), 41% (TALK score = 1–2), and 6% (TALK score = 3–4). The differences were significant (*p* < 0.0001). Although being simple to use, it is worth to mention some potential limitations of this score. Only 56% of the patients in the training cohort had laryngeal, and the remaining were diagnosed with hypopharyngeal or oropharyngeal primaries. Although externally validated with the Veterans Affairs Laryngeal Cancer Study data [67,68] purely consisting of laryngeal cancer, the performance of the model may allow room for improvement. All patients were treated with induction chemotherapy. This may limit the model’s use in the concomitant chemoradiotherapy setting. Even in the induction chemotherapy setting, the performance of the model may be limited for current use, due to the fact of outdated chemotherapy regimens and radiotherapy techniques, which were used in the training (1988–1995) and validation (1985–not provided) cohorts.

A more recent laryngectomy-free score (LFS) by Wichmann et al. [86] was developed using data of 52 patients treated in one center participating in the DeLOS-II trial (total number of patients included in the multicenter trial: 173) [80]. This score can be used three weeks after one cycle of docetaxel, cisplatin ± 5-fluoruracil ± cetuximab for a laryngeal or hypopharyngeal primary. It requires pre- and post-induction chemotherapy endoscopy and ^18^FDG-PET/CT examinations. The LFS score is the sum of the each of the following parameters with their corresponding rounded hazard ratio: 12 points for *n* = 3 (UICC 7^th^ edition), six points for > 0.2 residual CT-based tumor volume ratio (post-chemotherapy/baseline), five points for absolute residual CT-based tumor volume > 5.6 mL, and four points for residual maximum standard uptake value/residual mean standard uptake value (resSUVmax/resSUVmean) > 1.51 in ^18^FDG-PET/CT examination. An LFS > 16 was found to be predictive for significantly worse laryngectomy-free (*p* = 0.0014), overall (*p* = 0.0146) and tumor-specific survival (*p* = 0.0006). Although exciting, the model requires external validation and raises questions about its widespread application including low-cost healthcare settings or in countries/institutions where upfront concomitant chemoradiotherapy is preferred. For the latter, a predictive model able to differentiate patients at the time of initial diagnosis still does not exist. Nevertheless, the single-cycle induction chemotherapy approach with the use of endoscopic reevaluation, even in the absence of high-tech imaging, may save patients who are unsuitable for larynx preservation approach from the unnecessary toxicity and treatment delay caused by subsequent cycles [87].

On the other hand, even in the prospective randomized trial setting of DeLOS-II, only 57.4% of the poor responding patients agreed to undergo the indicated total laryngectomy [80]. This suggests the redundancy of an induction chemotherapy-based decision-making process even in the presence of any predictive models, if the patients are not properly pre-informed about the algorithm and the planned consequences of a responsive vs. a non-responsive tumor. Therefore, in institutions where the induction chemotherapy is the preferred standard over upfront chemoradiotherapy, the latter approach should be considered for patients who do not clearly agree to undergo a laryngectomy in case of a non-response to induction chemotherapy.

In T2Nany, selected T3Nany and even “early” T4Nany laryngeal cancers, an organ-sparing alternative to total laryngectomy, the concept of laryngeal organ preservation surgery by open and transoral robotic surgical techniques has been developed and may be applicable [88,89,90]. The review article by Succo and Crosetti (2019) showed, that in patients with early and intermediate T2–3 stages treated by open laryngeal organ preservation surgery, the five-year local control rates were above 90%, the five-year disease-free survival between 70% and 90% and the five-year overall survival 79.9% [88]. Different retrospective studies including patients affected by T3–4 laryngeal cancer and treated by laryngeal open preservation surgery showed a five-year loco-regional control, disease-free survival and overall survival rate of 82.9–96.2%, 78.2–87.9% and 82.2–87.8% in T3 and 51.4–71.7%, 49.0–68.1% and 71.2–73.7% in T4, respectively [51,91,92,93]. Five-year laryngeal function preservation and laryngectomy-free survival were 83.9–94.2% and 93.1% in pT3, and 59.3–78.0% and 75.5% in pT4, respectively [51,91,92,93].Without and statistical comparison involved, just by comparing the rates, these results are comparable, and even better, than the outcome of the studies comparing surgical and non-surgical treatment strategies showed in Table 2, which may also be due to a selection bias (patients in better health and performance status, selected tumors, etc.). No comparative studies were found in the current literature, making any conclusions for both, T3 and T4 laryngeal cancers, difficult.

Transoral robotic surgery for laryngeal cancer has been shown to be feasible for minimally invasive partial laryngectomy for either supraglottic or glottic cancer, as well as for total laryngectomy, in selected T1-3 patients [90]. However, the level of evidence for oncologic outcome as compared with other treatment modalities (open surgery, radiotherapy) remains low due to the small number of published series and the lack of randomized studies. Until today, it cannot be clearly stated that transoral robotic surgery results in oncologic and functional outcome similar to transoral laser or open organ preservation surgery. Further research is required to identify predictive markers for more accurate differentiation of patients suitable for organ-preservation strategies. It is more desirable to have an algorithm that can indicate the larynx-preservation candidates prior to the initiation of any treatment, including induction chemotherapy. Morphologic, metabolic and texture-based imaging criteria, molecular factors or a multimodal combination of these pose a likely future scenario [94,95]. There is another inevitable dilemma concerning the generation of such predictive models. The advantage of using the data of patients enrolled in prospective trials is to minimize known and unknown confounder effects. On the other hand, the profile of prospective trial patients does not mirror the broader spectrum patients seen on a daily basis.

### 3.2. T3N0-3

#### 3.2.1. Oncologic Outcome

There is a generally acknowledged equipoise regarding the surgical and non-surgical modalities for T3 glottic laryngeal cancer (Table 2). As an example, the adjusted risk models on the Surveillance, Epidemiology, and End Results (SEER) data which encompassed the period of 1999–2007 showed superior overall survival with primary total laryngectomy compared to primary chemoradiotherapy on elderly (> 65) patients diagnosed with stage III–IVB laryngeal cancer (*n* = 759). However, the results of the models lost their significance when the T4 primaries were excluded [96]. It is also worth to note, that the SEER does not report on the details of chemotherapy (e.g., concomitant or sequential). Later, another comparison of primary surgery and radiotherapy on T3 N0 laryngeal cancer based on a National Cancer Database (United States of America) cohort (*n* = 2622) was published by Ko et al. [97]. Unadjusted and adjusted five-year overall survival rates were comparable (adjusted: 53% and 54% after primary surgery and radiotherapy, respectively, *p* = 0.41). Rather than the selected primary treatment modality, increasing age, presence of co-morbidities, non-private insurance, < 70 Gy radiation dose and normofractionated radiotherapy alone were associated with impaired survival. More recently, Bates et al. [98] performed an “apples-to-apples” analysis by comparing the patients receiving the full dose of the planned treatment. Although showing superior overall survival after primary chemoradiotherapy vs. primary surgery in the univariate analyses, the statistical significance of treatment modality was lost in the multivariate analyses. Similarly, no survival benefit with one treatment modality to another in N0 or N+ subgroups could be demonstrated, but Timmermans et al. [99] showed an impaired five-year overall survival in T3N+ compared to T3N0 tumors. Smaller retrospective chart reviews on patients treated within the last two decades corroborate these findings [99,100,101].

The study by Timmermans et al. [99] compared survival according to staging (T3 vs. T4) and to treatment modality (total laryngectomy with adjuvant radiotherapy vs. chemoradiotherapy) and showed no difference for either of the two. However, the majority of T3 tumors were treated with organ-preserving chemoradiotherapy and the majority of T4 tumors were treated with total laryngectomy and adjuvant irradiation.

Although partial laryngectomy may be indicated in some T3 glottic laryngeal cancer, we could not find any study explicitly comparing the oncologic outcome of this type of surgery alone to chemoradiotherapy without mixing in patients treated with total laryngectomy and radiotherapy for the period after 1999 [97,100]. There are not enough data for transoral robotic surgery for this tumor stage until today.

#### 3.2.2. Laryngeal Preservation and Functional Outcome

We were not aware of any study comparing laryngo-pharyngeal function after surgical and non-surgical treatments exclusively in patients with T3 glottic laryngeal cancer treated within the last 20 years (Table 2). Laryngo-esophageal dysfunction-free survival at two years was shown in 40% of T3 tumors treated by chemoradiotherapy in the study of Timme et al. [100]. Twelve percent of the patients undergoing chemoradiotherapy were feeding-tube dependent at two years after treatment. Concerning the laryngeal preservation and function, they (*n* = 25) reported 44% laryngectomy-free and 52% tracheostomy-free survival at two years after chemoradiotherapy [100]. Of all survivors, 24% were tracheostomy-dependent two years after irradiation. In the whole cohort (74% T3, 26% T4), the overall larynx preservation after chemoradiation was 79%. In another small cohort (*n* = 18), a 43% two-year organ-preservation rate was reported by Bussu et al. [101]. Although a few patients were treated with partial laryngectomy in both cohorts, these rates are not provided in both articles.

Aspiration is one of the most important issues after radiotherapy to larynx. A retrospective study from MD Anderson Cancer Center (*n* = 40) reported on modified barium swallow results of patients after irradiation [102]. The majority of patients had locally advanced laryngeal cancer (60% T3, 15% T4). Eighty-four percent of patients aspirated, 44% being silently. Silent aspiration was more prevalent one year or later after the treatment. Sixty-eight percent reported dysphagia prior to treatment. With a median time of 22 weeks, eventually 48% of the feeding tubes were removed. This rate was 72% in the disease-free group.

### 3.3. T4aN0-3

#### 3.3.1. Oncologic Outcome

Compared to larynx-preservation modalities, primary surgery followed by adjuvant treatment indicates better overall survival based on most [98,99,101,103,104] but not all [100,105] retrospective cohort studies (Table 2). The key discussion in this stage is rather about the survival benefit through total laryngectomy weighted against its outcome in quality of life [106], especially in terms of a number-needed-to-treat perspective.

Regarding oncologic outcome, Grover et al. [103] analyzed the National Cancer Database (United States of America) data of 616 patients diagnoses with T4a laryngeal cancer. Around two-thirds were treated with chemoradiotherapy. Median overall survival was 61 vs. 39 months after primary surgery followed by adjuvant treatment vs. primary chemoradiotherapy (*p* < 0.001). Due to the nature of this registry-based study, no other outcome parameters could be reported. Nevertheless, the distribution of patient and treatment characteristics need to be highlighted. Patients with advanced nodal disease (N2 vs. N0: 26.6% vs. 43.4%, *p* < 0.001) and supraglottic (vs. glottic) location (31.3% vs. 47.5%, *p* < 0.001) were less likely to undergo total laryngectomy. Similarly, more patients were likely to be treated with primary surgery in high than low case-volume centers (46.1% vs. 31.5%; *p* < 0.001). The impact of high vs. low case-volume as well as academic vs. community centers on overall and progression-free survival is reported to be clinically prominent and statistically significant [107,108,109,110]. Another related issue is the fact that patients with significantly higher income, privileged ethnic background, better socio-cultural and economic status are prone to be offered surgery and these factors are shown to be associated with survival [105,111]. Although speculative, these data indicate that in most low case-volume and/or community hospitals, head and neck cancer patients may be treated by “general” rather than “specialized” radiation oncologists.

Concerning the presence or absence of accompanying nodal involvement, Bates et al. [98] found that the whole cohort of T4a patients benefited from primary surgery compared to primary chemoradiotherapy in terms of overall survival (48.9% vs. 39.4%; *p* < 0.01). However, the model lost its statistical significance, when the N0 patients were excluded. This indicates that the survival benefit of surgery may be set back by the presence of nodal involvement.

An interesting retrospective National Cancer Database (United States of America)-based study by Stokes et al. [105] compared three treatment modalities against each other in patients with T4a laryngeal cancer: primary surgery by means of total laryngectomy followed by adjuvant radiotherapy (TLE + RT), concurrent chemoradiotherapy (CRT) and multiagent induction chemotherapy starting 43 to 98 days before radiotherapy followed by radiotherapy analogous to PARADIGM [112] and DeCIDE [113] trials (IC + CRT). After adjusting for potentially confounding variables, CRT resulted in inferior overall survival compared to IC + CRT (hazard ratio [HR], 1.28; 95% confidence interval [CI]: 1.10–1.49; *p* < 0.01) and TLE + RT (HR, 1.48; CI: 1.32–1.65; *p* < 0.01). However, no survival advantage of TLE + RT was demonstrated over IC + CRT (HR, 0.87; 95% CI: 0.73–1.03; *p* = 0.10). Furthermore, the survival advantage of TLE + RT over CRT was lost when patients without adjuvant RT after surgery were included in the surgical cohort.

According to the definition used by the Radiation Therapy Oncology Group 91-11 trial, a “high-volume” T4a primary indicates a laryngeal cancer penetrating through the cartilage or extending more than 1 cm into the base of the tongue [72]. A more recent study by Hsin et al. [114] investigated this issue from a volumetric perspective. In their retrospective study with a modest cohort size (concomitant chemoradiotherapy: *n* = 48, total laryngectomy: *n* = 14), they isolated tumor volume ≥ 15 cm^3^ to be an independent poor prognostic factor in terms of loco-regional control, overall and progression-free survival, when treated with chemoradiotherapy compared to total laryngectomy.

Besides the fact, that primary surgery followed by adjuvant treatment indicates better overall survival, Timmermans et al. showed an impaired five-year overall survival in T4N+ when compared to T4N0 tumors [99].

Although partial open or transoral robotic laryngectomy may be indicated in some selected “early” T4 laryngeal cancer, we could not find any study explicitly comparing the oncologic outcome of these types of surgery alone to chemoradiotherapy.

#### 3.3.2. Laryngeal Preservation and Functional Outcome

Studies reporting larynx preservation and functional status exclusively for T4a primaries in our pre-defined timeframe are of very limited in size and are of a retrospective nature (Table 2). According to Bussu et al. [101], organ preservation after chemoradiotherapy (*n* = 10) was 17% after two years. Timme et al. [100] reported a two-year laryngo-esophageal dysfunction-free survival of 33% after chemoradiotherapy (*n* = 9). Sixty-six of the survivors at two years could preserve their larynx after chemoradiotherapy. Of all survivors, 17% were tracheostomy-dependent two years after irradiation. Astonishingly, no patient was feeding-tube dependent two years after an organ-preserving treatment strategy. Another retrospective study by Vengalil et al. [104] demonstrated a two-year tracheostomy-dependency rate of 55%, and a 3-year laryngectomy-free survival of 67% in a modest-sized cohort (*n* = 65) treated by radiotherapy with or without chemotherapy.

## 4. Summary

Surgical and radiation delivery techniques are evolving [28,30,31,34,90,115,116,117], and new systemic agents are improving the efficiency of locoregional treatments with an impact on survival [75,118,119]. Therefore, we tried to focus on published literature that compared surgical and non-surgical treatment strategies and was restricted on patients treated within the last two decades. Nevertheless, substantial numbers of patients included in these studies were not treated with contemporary techniques and modalities that have been widely used in the last decade. The oncologic and functional equipoise between transoral laser surgery and radiotherapy in T1–2 stage glottic laryngeal cancer was demonstrated in previously published meta-analyses [6,7,8,24,25,120]. The recent evidence falling in our pre-defined timeframe corroborates these findings. On the other hand, the recent evidence for the management of locally advanced glottic laryngeal cancer indicates room for improvement concerning the ideal selection of patients for optimal laryngo-esophageal dysfunction-free and overall survival with tailored multimodal treatment.

Also emphasized by Beitler et al. [106], patients with better socio-economic backgrounds are considered better candidates for primary surgery. Moreover, concerning the issue of selection bias, comorbidities are better documented in hospitalized patients (surgery) than in patients treated on an outpatient basis (radiotherapy), due to financial incentives for reimbursement. Regarding logistic hassle for patients, surgical treatment is short, one-stop and therefore performable in “centers of excellence” for those who have to travel long distances. On the contrary, radiotherapy is usually performed in an ambulatory setting, which takes weeks, and the public as done for more mainstream medical concepts such as surgery does not perceive the importance of its quality. This leads many patients opting for low-case volume, non-academic centers close to their homes. Despite of these disadvantages—and the fact that more T4a patients are receiving non-surgical treatments—the overall and disease-specific survival gap between surgical and non-surgical modalities in locoregionally advanced laryngeal cancer has almost closed within the last three decades [106,121].

Nevertheless, current evidence does not clearly indicate an ultimate superior oncologic outcome with chemoradiotherapy compared to partial/total laryngectomy and adjuvant treatment in T3-4a laryngeal cancer. A large body of evidence indicates the futility and inferiority of results of organ preservation in extensive T4a disease, where surgical salvage may not be offered as a safe back-up plan in case of treatment failure. On the other hand, a larynx-preserving primary radiation strategy seems to be comparable to partial/total laryngectomy, followed by adjuvant treatment for T3 and limited for well-functioning, properly selected T4a cases.

The lack of high-level evidence comparing contemporary open or transoral robotic organ-preserving surgical (rarely T1, T2, selected T3 and “early” T4) and non-surgical modalities does not allow any concrete conclusions to be drawn in terms of oncologic and functional outcome. However, these surgical strategies may be a good option in selected cases, and in the hands of experienced surgeons, to preserve the larynx and avoid or deintensify (chemo)radiotherapy. The dose and volume differences between primary and adjuvant radiotherapy are usually minimal. Therefore, patients requiring an adjuvant radiotherapy after partial laryngectomy will be exposed almost to the same toxicities similar to a primary radiotherapy. Because of this, partial laryngectomy should be only indicated for patients with a chance to omit postoperative radiotherapy. This lack of high-level evidence comparing contemporary surgical (e.g., open organ-preserving surgery, transoral robotic surgery) and non-surgical modalities supported by modern imaging techniques needs to be addressed with properly designed clinical trials and adequate funding.

## 5. Conclusions

The findings and the discussion can be concluded with the following recommendations: **T1-2N0M0 glottic laryngeal cancer:** (1) Based on the equipoise of oncologic and functional outcome after transoral laser surgery and radiotherapy, informed and shared decision-making with—and not just about—the patient poses a paramount importance. (2) Studies utilizing modern imaging modalities and comparing contemporary transoral surgical and more precise irradiation techniques are required. **T3-4aN0-3M0 glottic laryngeal cancer:** (1) There is a generally acknowledged equipoise regarding the surgical (partial/total laryngectomy) and non-surgical modalities for T3 glottic laryngeal cancer. (2) Patients with extensive and/or poorly functioning T4a laryngeal cancers should not be offered organ-preserving chemoradiotherapy with the false hope of salvage surgery as a back-up plan, but instead, total laryngectomy with adjuvant (chemo)radiation. (3) Unnecessary tri-modality treatments should be avoided if an equally successful uni- or bi-modality therapy is available (e.g., partial laryngectomy for T3 tumors, if an aggressive adjuvant treatment cannot be omitted). (4) In order to identify patients who would benefit from larynx preservation, externally validated predictive markers and algorithms are needed. (5) Instead of offering one-size-fits-all approaches and overly standardized rigid institutional strategies, patient-centered, informed and shared decision-making should be favored. (6) While being aware of the lack of supporting evidence, open or transoral robotic organ-preserving surgery may be implemented by experienced head and neck surgeons for selected cases, especially if the avoidance of adjuvant (chemo)radiotherapy seems to be realistic.

## Figures and Tables

**Table 1:** Various five-year outcome parameters of early stage glottic laryngeal cancer (T1-2N0) according to T stage.

Stage	Local Control	Disease-Specific Survival	Disease-Free Survival	Overall Survival	Voice	Laryngeal Preservation	Comment
	%						
T1a	RT: 93 TOLS: 81 [14]	RT: 96 TOLS: 100 [14]	RT: 78 TOLS: 69 [14,15]	RT: 86–89 TOLS: 86–87 [14]	Significantly better VHI, GRBAS, jitter, shimmer, noise-harmonic rate and maximum phonation time after RT (1) [14,16]	RT: 77–93 TOLS: 69–100 [14]	No significant difference except for (1)
T1b	RT: 86 TOLS: 95 in 2 years * [17]		RT: 86 TOLS: 89 in 2 years * [17]	RT: 95 TOLS: 94 in 2 years * [17]	Significantly better VHI and less voice deficiency after RT (1) [14]	RT: 86 TOLS: 100 in 2 years * [17]	No significant difference except for (1)
T1a-b mixed	RT: 87-94 TOLS: 75–91 (1) [5,14,18]	RT: 96–97 TOLS: 100 [5,14]	RT: 87% TOLS: 75% (1) [5]	RT: 79–89 TOLS: 86–91 [5,14,18]	Significantly better understandability and VHI scores after RT (2) [14,19] No difference (3) [18] Varied (4) [20]	RT:93–94 TOLS: 100 [14,18]	No significant difference except for (1): one study [5] showed better LC and DFS after RT. (2): Better understandability and VHI scores after RT (3): One study [18] showed no difference. (4): One study [20] showed worse GRBAS if cordectomy > type II
T2	RT: 76 TOLS: 88 in 3 years * [21]		RT: 71 TOLS: 88 in 3 years * [21]			RT: 94 TOLS: 100 in 3 years * [21]	No significant difference despite selection bias
T1-2 mixed	RT: 86 TOLS: 75 [14]	RT: 94 TOLS: 99 [14]		RT: 72 TOLS: 90 [14]	Significantly better VHI and understandability after RT(1) [14,19]	RT: 76-83 TOLS: 87-93(2) [14,19]	No significant difference except for (1) and (2): One study [14] showing better (93% vs. 83%) laryngeal preservation after TOLS, whereas RT was more often offered for advanced stages.

*: No study with five-year outcome found that compared treatment modalities on patients exclusively treated in and after 1999; DFS: disease-free survival; GRBAS: grade (G), roughness (R), breathiness (B), asthenia (A), and strain (S); LC: local control; RT: radiotherapy; TOLS: transoral laser surgery; VHI: voice handicap index.

**Table 2:** Various five-year outcome parameters of locally advanced glottic laryngeal cancer (T3-4aN0-3) according to T stage.

Stage	Loco-Regional Control	Overall Survival	Laryngeal Preservation	Comment
**T3N0-3**		CRT: 45–61 RT: 35–51 Surg.: 52 Surg. + RT: 54 Surg. + CRT: 50 TLE + (C) RT: 46–53 [97,98,99,100]	CRT: 43–65 in 2 years * [100,101]	Equivalent OS among all compared groups OS: T3N0 > T3N+ (65 vs. 35, sign.) [99]
**T4N0-3**	CRT: 56.2 TLE + CRT: 86.6 [80]	Ind. CX + CRT: 45 CRT: 33–37 RT: - Surg.: - Surg. + RT: - Surg. + CRT: - TLE + (C) RT: 48.9–58 [75,80,81,82]	CRT: 17–66 in 2 years * [100,101]	Significant better LRC and OS after primary TLE than CRT OS: T4N0 > T4N+ (58 vs. 35, sign.) [99]

*: No study with five-year outcome found that compared treatment modalities on patients exclusively treated in and after 1999; CRT: chemoradiotherapy; LRC: loco-regional control; OS: overall survival; RT: radiotherapy; Surg.: partial or total laryngectomy; TLE: total laryngectomy.
